# Comparison of different critical nitrogen dilution curves for nitrogen diagnosis in rice

**DOI:** 10.1038/srep42679

**Published:** 2017-03-06

**Authors:** Syed Tahir Ata-Ul-Karim, Yan Zhu, Xiaojun Liu, Qiang Cao, Yongchao Tian, Weixing Cao

**Affiliations:** 1 National Engineering and Technology Center for Information Agriculture, Jiangsu Key Laboratory for Information Agriculture, Jiangsu Collaborative Innovation Center for Modern Crop Production, Nanjing Agricultural University, 1 Weigang Road, Nanjing, Jiangsu 210095, P. R. China

## Abstract

The critical nitrogen (N) dilution curve is a suitable analytical tool for in-season estimation of N status to implement precision N management. This study was undertaken for a comprehensive comparison of N dilution curves in Japonica and Indica rice to investigate, whether a single curve can be used for both rice ecotypes and to determine the most robust plant index for assessing N status in rice ecotypes. The different N dilution curves were developed based on plant dry matter (PDM), leaf area index (LAI), leaf dry matter (LDM) and stem dry matter (SDM) for N diagnosis in Japonica and Indica rice. The comparison of N dilution curves of two rice ecotypes showed non-significant differences, therefore a single/unified curve can be used to assess plant N status for precision N management in both rice ecotypes. The relationships between PDM based, with LAI, LDM, and SDM based N nutrition index, accumulated N deficit and N requirement, indicated that leaf based approaches could be used as substitutes for PDM approach. The lower coefficient *b* values of LDM based curve (due to efficient physiological N use in leaves) implied that LDM was the most appropriate approach for developing N curve as compared to other approaches.

Nitrogen (N) is the most important nutrient for crop plants. Its significance for crop production is associated with crop N uptake and growth response. Understanding the processes governing the N uptake and its allocation in different plant organs, is of major importance with respect to crop productivity and environmental sustainability^1^. Under sub-optimal N nutrition, the N uptake rate varies significantly, depending upon the crop growth stage, cropping site, and year. However, under ample N supply, crop N uptake directly controls crop growth rate, dry matter (DM) accumulation and leaf area (LA) expansion, and is regulated by several internal plant mechanisms^2^. The concept of critical N concentration in plant tissues has been defined as, the minimum N concentration essential to attain the maximum crop growth rate^3^. Tissue N concentration varies in different plants organs during crop growth period^4^. Several approaches, such as DM accumulation, LA expansion and crop growth stage have been used to derive the N concentration and its change over crop growth progress^1^,^2^,^5^. N concentration declines during the crop growth period, however, the relationships of DM accumulation and LA expansion with N concentration remain similar in major cultivated species of C_3_ and C_4_ plants^2^.

The critical N concentration due to its ontogenetic decline with crop growth, is derived as a negative power function called “dilution curve”. This concept has been established for over two decades and N dilution curves have been developed in various crop species, including wheat^6^,^7^ and rice^8^,^9^ on plant dry matter (PDM) basis as well as on LAI^10^,^11^ and specific organ (leaf, stem, and spike) DM basis^12^,^13^,^14^,^15^. Additionally, this concept has been successfully used for in-season estimation of N requirement, grain yield and setting yield targets according to different N application rates in rice ecotypes^1^,^5^. However, it is destructive and time-consuming. Therefore, attempts have been made to integrate the concept of critical N curve with chlorophyll meter readings to develop an effective and non-destructive approach for N diagnosis in rice^16^.

Plant growth is the sum of metabolic and structural compartments, each having its own demand for metabolic and structural N^17^. The development of N dilution curves on DM of specific organs and LAI basis, rather than classically used PDM based approach and their comparison with each other, is not only essential for an in-depth and improved understanding of this concept, but also crucial for finding the genotypic and environmental differences of crop N dynamics in a physiologically functional manner as well as for developing crop simulation models^18^. Classically used PDM approach offers sufficient insight into the factors governing N uptake in rice. However, the variations, associated with the high degree of in-season spatial distribution of crop N status within the crop field, restrict the adaptation of PDM approach in modern mechanized agricultural practices^19^. LAI, being a fundamental variable in agricultural studies, has been successfully used for monitoring crop growth, yield prediction, and agronomic management optimization. Its estimation can be done using destructive and non-destructive methods, but the latter are usually preferred for their relative accuracy and convenience. Remote sensing technologies can also be used for the estimation of PDM directly, yet their capability to accurately estimate PDM is limited, as the recorded spectral responses are mainly related to the interaction between the sun radiance and plant canopies^10^,^11^. Thus, the correlation between PDM and spectral responses or vegetation indices is usually poor under closed canopy for which spectral responses become saturated and lose sensitivity to PDM^10^. Additionally, the partitioning of DM among different plant organs influences the PDM/N and LAI/N relations and causes changes in the shape of dilution curve, thus limits their acceptance as reliable methods^4^. The real-time, rapid and non-destructive field methods generally monitor N concentration at a single leaf or on canopy basis. However, single leaf based diagnosis of N status in crops is exaggerated by progressive shading from newer leaves, a decline of leaf N concentration from aging and abiotic stress, as well as an increase in the proportion of structural tissues^20^. Both stem dry matter (SDM) and leaf dry matter (LDM) significantly contribute towards PDM during vegetative phase, yet SDM is considered the most N dilution determining factor for the entire plant, mainly because of being significantly higher than that of LDM^21^.

Despite the pros and cons of aforementioned approaches, an integrated investigation of these approaches and their comparison could give useful insight into plant N status, which has not been yet attempted in any crop including rice. Moreover, the gradual decreases in N uptake and use efficiencies, due to selection of N responsive high yielding cultivars in past few decades, resulted in significant differences between the N dilution curves developed in recent years and those developed in past. Therefore, the rigorous updates of these algorithms are urgently required for better understanding of this concept.

The objectives of this study were to compare the N dilution curves in rice ecotypes on different bases to determine the differences among them and to assess the possibility to develop a single curve of Indica or Japonica rice to be used under different climatic conditions. Moreover, the study tried to investigate the most reliable and robust plant basis or approach for in-season estimation of rice N status. This study represents the first ever most comprehensive comparison of critical N curves in different ecotypes and plant bases in any crop. The results of this study will provide an integrated methodology for diagnosing plant N status and guidance for precision N management at critical growth stages in different crop ecotypes, thus contribute towards the sustainability of intensive agricultural ecosystems globally.

## Results

### Critical, maximum and minimum N dilution curves on difference bases

**Figure 1:**
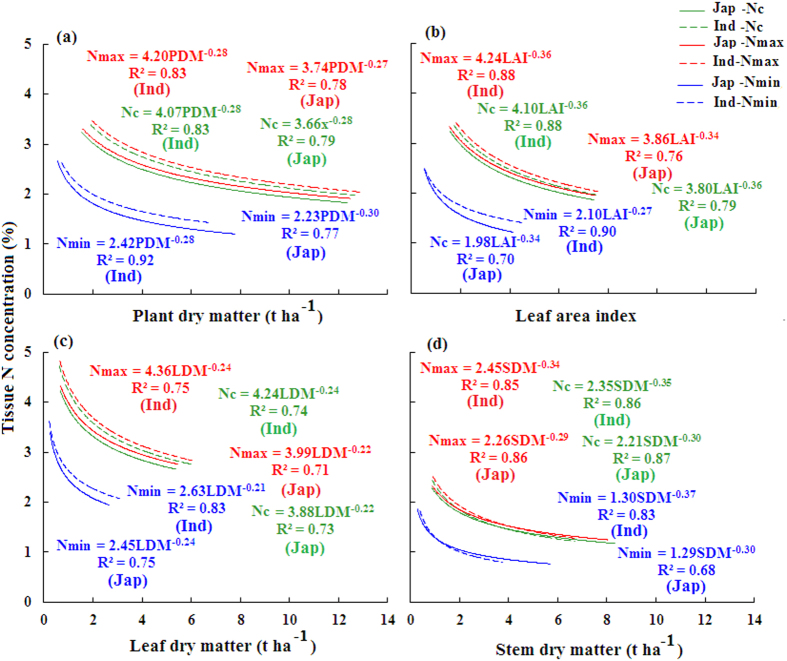
Comparison of critical, maximum and minimum N dilution curves obtained by non-linear fitting for Japonica and Indica rice on different bases in eight varied N rates experiments (**a**): plant dry matter basis; (**b**) leaf area index basis; (**c**) leaf dry matter basis; d stem dry matter basis).

**Figure 2:**
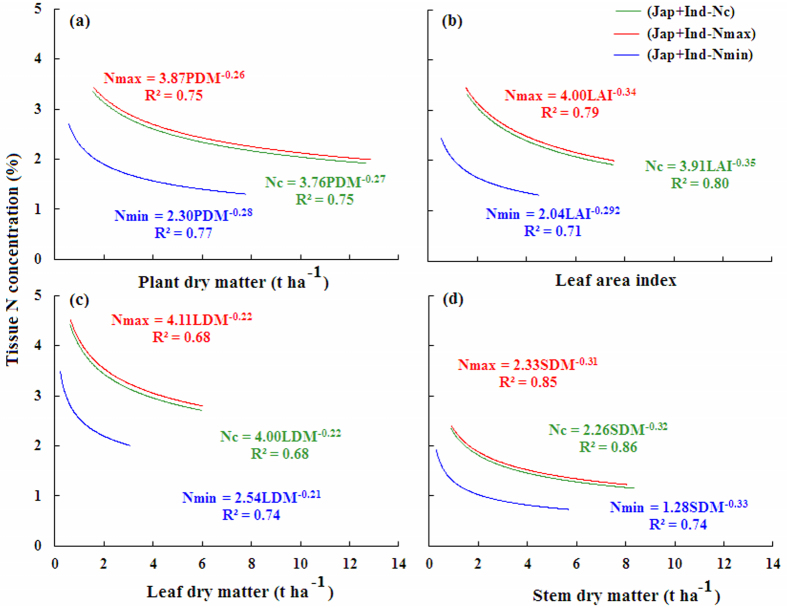
Comparison of critical, max and minimum nitrogen dilution curves obtained by non-linear fitting for Japonica + Indica rice on different bases in eight varied N rates experiments (**a**) plant dry matter basis; (**b**) leaf area index basis; (**c**) leaf dry matter basis; (**d**) stem dry matter basis).

As shown in Fig. 1, PDM, LAI, LDM and SDM data that fit the statistical criteria for development of N dilution curves, respectively, ranged from 1.55–12.37 t ha^−1^, 1.57–7.41, 0.71–5.37 t ha^−1^, and 0.88–8.33 t ha^−1^ for Japonica rice, whereas 1.91–12.67 t ha^−1^, 1.80–7.52, 0.63–5.98 t ha^−1^, and 0.90–6.69 t ha^−1^ for Indica rice. The coefficients *a* and *b* of the N dilution curves based on PDM, LAI, LDM and SDM varied, respectively, from 2.21 to 3.88 and 0.22 to 0.36 in Japonica rice, while from 2.35 to 4.24 and 0.24 to 0.36, respectively in Indica rice (Fig. 1). The R^2^ values for the fit of the curves through all the data points on different bases varied from 0.73 to 0.87 and 0.74 to 0.88 in Japonica and Indica rice, respectively (Fig. 1). The curves between the two rice ecotypes on different bases showed non-significant differences (F_calculated_ = 1.17, 1.29, 1.05 and 1.41 < F_tabulated (1-62)_ = 4.00, α = 5%), when tested according to method of Hahn^22^. Hence, there is a possibility to establish unified dilution curves on different bases by pooling the data of Japonica and Indica rice (Fig. 2). The unified dilution curves of Japonica + Indica rice can be used as potential alternative of single ecotype curve in intensive rice cropping systems, especially, where both rice ecotypes are cultivated.

Meanwhile, the upper limit (Nmax) and lower limit (Nmin) dilution curves on PDM, LAI, LDM and SDM bases in Japonica and Indica rice were determined using the data points not retained in the determination of critical N curves. The coefficients *a* and *b* of the Nmax and Nmin curves based on PDM, LAI, LDM and SDM varied, respectively, from 2.26 to 3.99, 1.29 to 2.45, and 0.22 to 0.34, 0.24 to 0.34 in Japonica rice, whereas from 2.45 to 4.36, 1.30 to 2.63, and 0.24 to 0.36, 0.21 to 0.37 in Indica rice (Fig. 1).

### Nitrogen nutrition index, accumulated N deficit and N requirement from different critical N dilution curves

**Figure 3:**
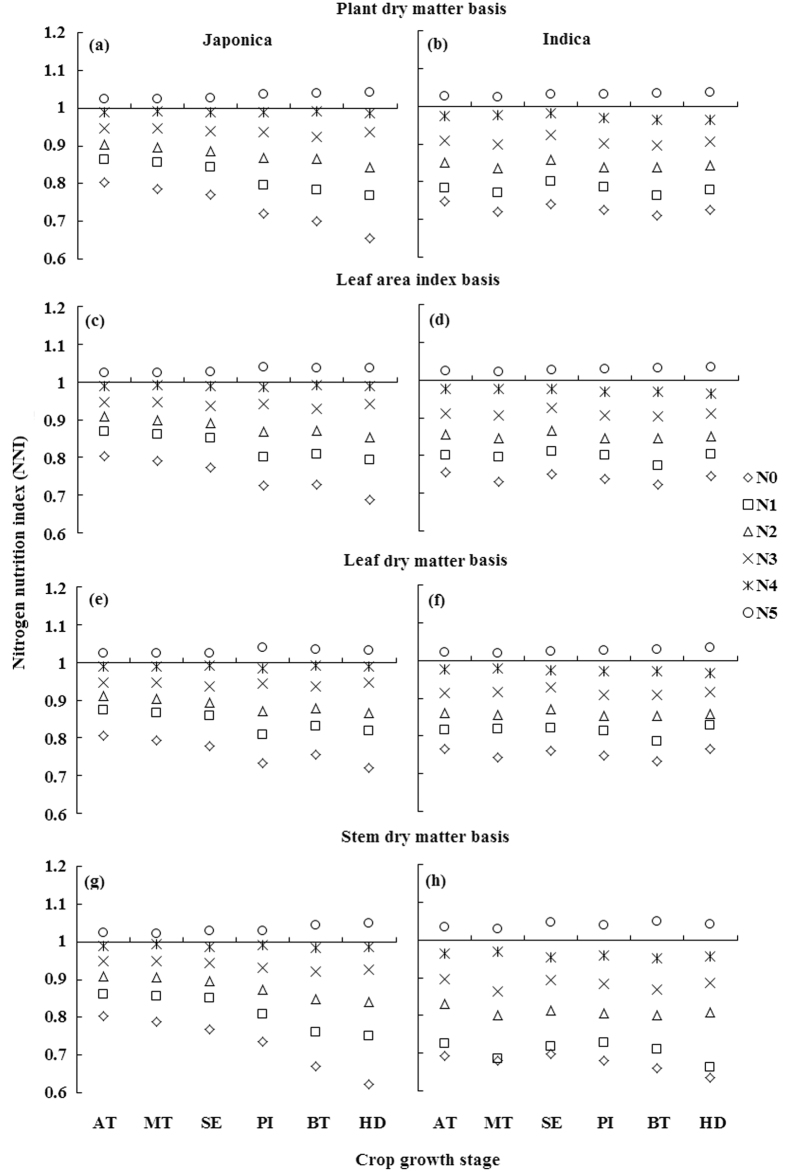
Nitrogen nutrition index (NNI) of Japonica and Indica rice at different growth stages in eight varied N rates experiments on the bases of different N dilution curves (**a,b**) plant dry matter basis; (**c,d**) leaf area index basis; (**e,f**) leaf dry matter basis; (**g,h**) stem dry matter basis). For X-axis, AT represents active tillering, MT mid tillering, SE stem elongation, PI panicle initiation, BT booting, and HD heading.

**Figure 4:**
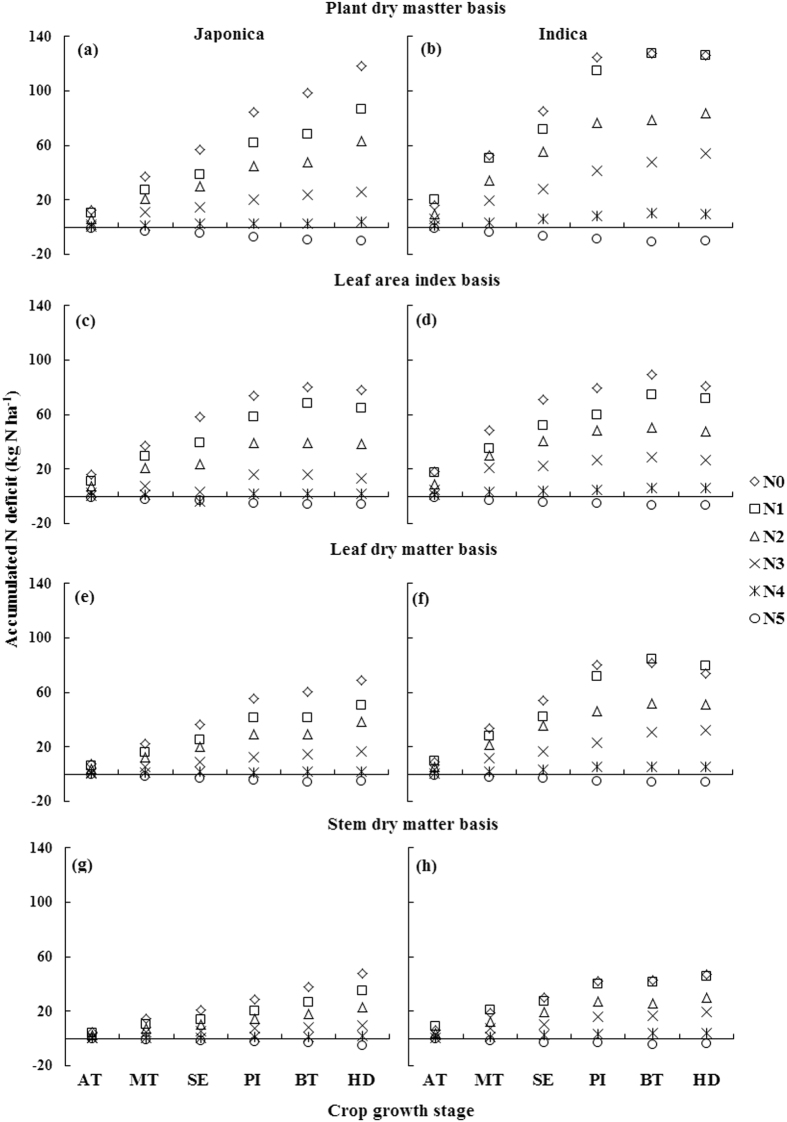
Accumulated N deficit (AND, kg ha^−1^) of Japonica and Indica rice at different growth stages in eight varied N rates experiments on the bases of different N dilution curves (**a,b**) plant dry matter basis; (**c,d**) leaf area index basis; (**e,f**) leaf dry matter basis; (**g,h**) stem dry matter basis). For X-axis, AT represents active tillering, MT mid tillering, SE stem elongation, PI panicle initiation, BT booting, and HD heading.

**Figure 5:**
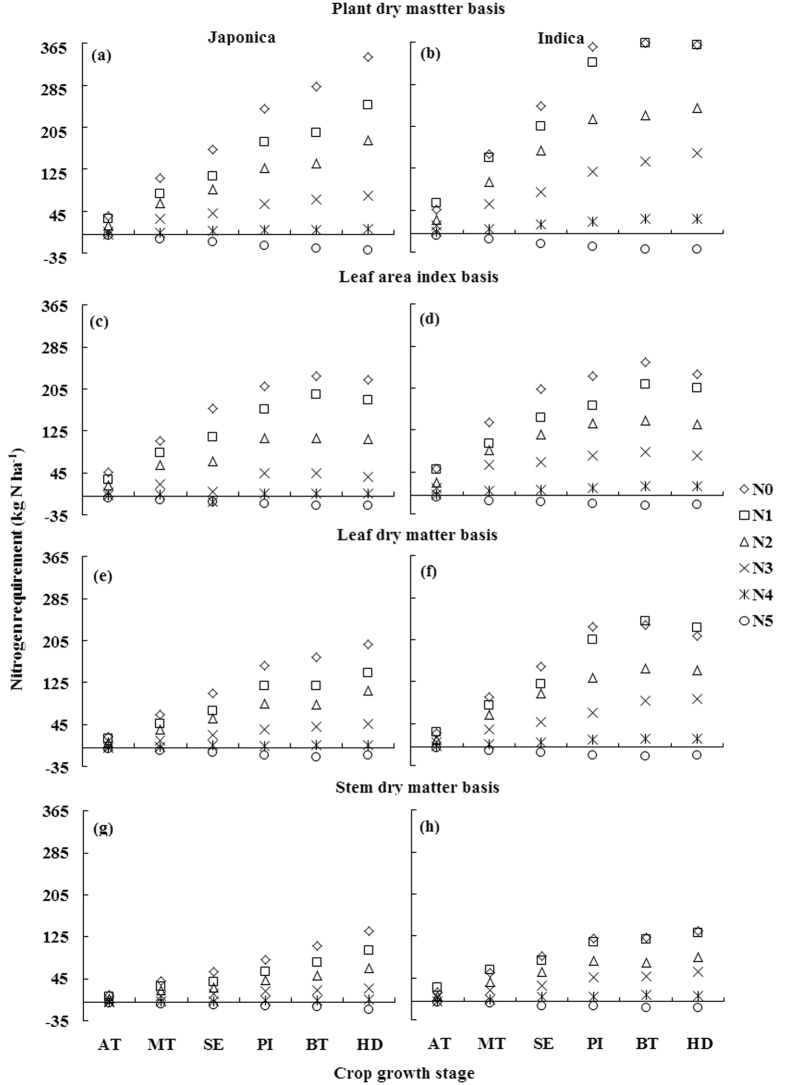
Nitrogen requirement (NR, kg ha^−1^) of Japonica and Indica rice at different growth stages in eight varied N rates experiments on the bases of different N dilution curves (**a,b**) plant dry matter basis; (**c,d**) leaf area index basis; (**e,f**) leaf dry matter basis; (**g,h**) stem dry matter basis). For X-axis, AT represents active tillering, MT mid tillering, SE stem elongation, PI panicle initiation, BT booting, and HD heading.

For any particular stage, the observed NNI (nitrogen nutrition index), AND (accumulated nitrogen deficit) and NR (nitrogen requirement) on the basis of different N curves in Japonica and Indica rice showed significant variations due to varied input levels of N fertilization. The values of NNI under sub-optimal N nutrition (N0, N1 and N2), optimal (N3 and N4) and under supra-optimal N nutrition (N5) were <1, around 1 and >1, respectively (Fig. 3), while that of AND and NR under similar conditions on the basis of different N dilution curves in two rice ecotypes were <0, around 0 and >0, respectively (Figs 4 and 5). The successful differentiation of the sub-optimal, optimal and supra-optimal N supply by estimated NNI, AND and NR confirmed their applicability as useful N diagnostic tools. Generally, the NNI under sub-optimal N supply decreased over the growth progress from active tillering (AT) to heading (HD) stage on the basis of different N dilution curves in two rice ecotypes. However, the decrease was till booting (BT) stage for PDM, LAI and LDM basis curves under sub-optimal N supply in Indica rice (Fig. 3b,d and f). Under optimal N supply, the minor decrease in NNI was observed till BT stage, followed by a slight increase in NNI at HD stage in both rice ecotypes. In contrast, the increase in NNI on different bases in rice ecotypes was observed under supra-optimal N supply. The appraisal of AND and NR on the basis of PDM, LDM and SDM curves in Japonica rice (Figs 4 and 5a,e,g) and on SDM basis in Indica rice (Figs 4 and 5h) increased from AT to HD stage, while that on PDM and LDM in Indica rice (Figs 3 and 4b,f) and on LAI basis in rice ecotypes (Figs 4 and 5c,d) increased from AT to BT stage, and then decreased. Similar trends of NNI, AND and NR were observed for the pooled data (Japonica + Indica) of both rice ecotypes on different bases (Supplementary Figs 1, 2 and 3).

### Relationships between plant dry matter based and leaf area index/organ based N parameters

This study further established the relationships between plant based and LAI/organ based N parameters in two rice ecotypes at different stages during vegetative growth to find out an alternative and most appropriate approach for in-season assessment of crop N status, instead of PDM approach (Supplementary Figs 4, 5 and 6). The LAI/organ based N parameters were expressed as a function of plant based N parameters. The R^2^ values for the relationships between plant based NNI and LAI/organ based NNI ranged from 0.9956 to 0.9996 and 0.9504 to 0.9995 for Japonica and Indica rice, respectively. These relations were robust, yet they were strongest on LAI basis, followed by LDM and SDM basis (Supplementary Fig. 4). The R^2^ values for the relationships between plant based AND, NR and LAI/organ based AND, NR ranged from 0.9597 to 0.9991 and 0.9505 to 0.9981 for Japonica and Indica, respectively. The plant and organ based relations were robust, with the best performance on LDM basis, followed by SDM and LAI basis (Supplementary Figs 5 and 6).

## Discussion

Nitrogen fertilizer application is one of the most important agronomic management strategies implemented for sustaining global food security over decades, and its management is the key to successful crop production. Understanding of N management in relation to N supply and demand as well as the underlying processes governing N uptake and distribution in plant organs is imperative for quantifying N dynamics in the cropping system^1^. N fertilization management in rice and other crops at key growth stages is a growing practice implemented for increasing N use efficiency. The N curves have been employed over two decades for precision N management in various crops. The N dilution curves have been developed for Indica and Japonica rice on PDM basis in independent studies conducted in different climates^8^,^9^, as well as on LAI and specific organ (leaf and stem) DM basis for Japonica rice^10^,^12^,^14^. This study was conducted to develop the N dilution curves for Japonica and Indica rice on PDM, LAI, LDM, and SDM basis, to compare these curves with existing ones for assessing N status in rice, and then to find out the most appropriate approach for in-season estimation of rice N status. However, according to Sheehy *et al*.^8^, their curve (existing curve of Indica rice) was developed by using the cruder method instead of classically used methodology proposed by Justes *et al*.^6^. The datasets used to develop the existing Indica rice curve were collected from the experiments, not initially designed for developing N dilution curve. The varying intervals between N application timing and plant sampling in developing the existing curve also resulted in overestimation. In contrast, the present study was conducted only to develop N dilution curves on different bases in rice ecotypes for their comprehensive comparison and integration for an in-depth understanding of this concept.

### Comparison of critical nitrogen dilution curves for Japonica and Indica rice

Rice cultivars are often classified into Japonica and Indica ecotypes and they differ from each other on the basis of morpho-physiological traits, origin, base temperature requirements, photosynthesis, net assimilation and respiration rates^23^. Indica rice is well adapted to tropical conditions, but it also thrives sufficiently in subtropical climates. However, the Japonica rice is adapted only to subtropical regions, and can be distinguished by its strong responsiveness to N fertilizer^24^. The present study developed critical N curves in Japonica and Indica rice to compare them with the existing ones to answer, whether or not a single N dilution curve of Indica or Japonica rice could be used both under tropical and subtropical conditions. The obvious divergences observed between the coefficients (*a, b*) of existing critical N curves for Indica rice in tropics (5.20, 0.50) and Japonica rice in sub-tropics (3.53, 0.28) (Table 1), as well as between the newly developed PDM based curves of Japonica (3.66, 0.28) and Indica rice (4.07, 0.28) in sub-tropics (Fig. 1a), were mainly attributed to differences in climatic conditions, rather than genetic variations between rice ecotypes because Indica rice is well adapted to tropical conditions, whereas Japonica rice is adapted to subtropical region^9^,^24^. The differences in coefficients (*a, b*) of newly developed critical N curve of Indica rice (4.07, 0.28) against the existing one (5.20, 0.50) were associated with their different growth rates under different climatic conditions. The lower coefficients (*a, b*) values of Indica rice grown in the subtropical region than those in tropics were attributed to slower crop growth rates and lesser tissue N requirements under lower temperatures^9^.

Apart from the aforementioned climatic, genetic and ecophysiological factors possibly contributing to these differences, timing and rate of N dressing application and the simple/cruder producer adopted for its development might also have contributed to these differences and cannot be overlooked. It has been established in earlier reports that too short or too long intervals between N application and plant sampling will ultimately lead to under or overestimation of N, mainly because of failure of PDM differentiation among N treatments due to variation in PDM upon increasing N application rates^17^. The time-series coevolution ensured monotonic N supply for regular N uptake and robust plant allometry. The existing N dilution curve of Indica rice in tropics were developed without considering the time-series coevolution between PDM-N with clear identification of the N application rates and timing. Therefore, the present study developed a new N dilution curve for Indica rice using a more systematic data set for comprehensive and robust comparison with existing and newly developed curves for rice. The PDM-N relations in present study were more reliable, because they were developed by considering the time-series coevolution between PDM-N with clear identification of the N application rates and timing.

In order to answer the original fundamental problem, whether or not a single critical N curve of Indica or Japonica rice could be used both under tropical and subtropical conditions, the critical N curve of Indica rice in tropics on LAI basis (3.22, 0.16) reported by Lemaire *et al*.^17^ was used. The coefficient *a* (3.22) of the curve reported by Lemaire *et al*.^17^, lies closer to the coefficient *a* of existing (3.53, 3.66 and 4.07) and newly developed (3.70, 3.80 and 4.10) N curves in rice ecotypes on PDM and LAI basis in subtropical climate. In contrast, the coefficient *a* of this curve lies very lower than that of (5.20) Indica rice in tropics, which ruled out the role of climatic conditions as a single major determining factor for these differences, and gave a possibility of using a single critical N dilution curve of Japonica or Indica rice developed under tropical or subtropical climatic conditions for in-season diagnosis of N status in rice. The differences in coefficient *b* of LAI based curve of Indica rice with other curves were attributed to low LA expansion in tropical climate because of shortened growth period in tropics.

### Comparison of critical nitrogen dilution curves with different bases

The newly developed and existing N dilution curves on four different bases in three rice groups were compared to explore their potential to assess N status in rice and then to find out the most reliable approach or approaches for in-season estimation of rice N status. The N dilution curves developed in the present study on four different bases were very close to those established previously on an individual basis (for each of PDM, LAI, LDM and SDM) in Japonica rice^9^,^10^,^12^,^14^ (Table 1). In the present study, the coefficients (*a, b*) of the PDM based curves (3.66, 0.28; 4.07, 0.28; 3.76, 0.27) were slightly lower (Figs 1a and 2a) than those on LAI basis (3.80, 0.36; 4.17, 0.36; 3.91, 0.35) in three rice groups (Figs 1b and 2b). The higher coefficients (*a, b*) values on LAI basis than that on PDM basis in rice ecotypes was mainly because of the ability of rice plant to accumulate more LAI than PDM at early growth stages^10^. However, towards advancing maturity, the coefficients of the critical N curve were affected due to self-shading of leaves as well as changes in LA ratios and leaf/stem ratios^3^.

The differences between coefficients *a* (3.88, 4.24 and 4.00) and *b* (0.22, 0.24 and 0.22) on LDM basis against those on PDM, LAI and SDM bases in three rice groups in the present study were attributed to interconnected photosynthetic properties of leaves and N concentration^25^, and indicated that rice plant exhibited different N accumulation rates among different plant organs during growth period at sampling dates. Moreover, leaf growth is a vital feature of plant N demand because of their role and higher requirements of reduced N in photosynthesis as compared to other plant organ^26^. The differences observed between the coefficients *a* of critical N curves in the present study on PDM and LAI basis were due to bi-compartmental (metabolic + structural) aggregation of N in plant organs, related to the weight/N in the entire plant. Furthermore, stress responses can also cause changes in the bi-compartmental partitioning of DM among plant organs, thus affected the shape of the dilution curves^4^. The coefficients (*a, b*) of critical N dilution curves on SDM basis in the three rice groups (2.21, 0.30; 2.35, 0.32; 2.26, 0.32) were lower than those on PDM, LAI and LDM basis in the present study (Fig. 1d and Supplementary Fig. 1d). The differences of SDM based curves against those on LDM based ones were primarily accredited to stem/leaf ratio. The decline in stem N during vegetative growth was associated with the decline in metabolic DM with high N contents, as well as increase in the proportion of structural and non-photosynthetic DM with low N contents. Thus, a greater fraction of structural DM in the stem than in leaves attributed to the differences between the LDM and SDM based curves. The observed differences between the coefficients *b* on LDM and SDM based curves in the three rice groups were directly related to the distribution of DM between leaves and stem. In contrast, the differences between coefficients *b* of the curves on SDM basis, compared with those of PDM basis, were negligible. Since, stem contributed more in the weight percentage in the total PDM, hence exerted a dilution effect on the total N in plant tissues. The coefficient b indicates the dilution intensity of critical N and is directly related to the dry matter partitioning between the plant organs (leaves and stems) during crop growth period. The lowest values of coefficient *b* in LDM based curves of all the three rice groups indicated that the most efficient physiological use of N occurred, when weight/N relations were established on LDM basis rather than PDM or SDM basis and were in agreement with the findings of Oliveria *et al*.^21^, that the lower values of coefficient b indicates the most efficient physiological use of N during crop growth period. The highest values of coefficients *b* in LAI based curves of all the three rice groups were attributed to leaf senescence at heading stages, which leads to over-estimation of coefficients *b* in the present study. The comparison of the parameters of curves on different plant bases is essential to illustrate organ specific N dynamics during crop growth and detailed responses to N deficiency and excess.

### Comparison of maximum and minimum nitrogen dilution curves with different bases

The Nmax and Nmin curves are referred as threshold N concentrations. There is a disagreement among researchers about the factors that control N uptake by crops under field conditions. For situation below the Nmax curve and above the N dilution curve, N absorption is determined by mineral N availability in the soil and is independent of the growth rate, while in the area below the N dilution curve and above the Nmin curve, N absorption is limited by mineral N availability in the soil and determines the growth rate^6^. Therefore, developing and comparing Nmax and Nmin dilution curves on different plant bases will offer the opportunity to calculate the degree of structural and metabolic N deficiency and excess in plant tissues to answer that what factors control N uptake by crops in field under different N supply conditions.

The Nmax and Nmin dilution curves on PDM, LAI, and SDM bases were lower than those on LDM basis in both the rice ecotypes. The higher values of the coefficient *a* and lower values of coefficient *b* on LDM based curves in the two rice ecotypes were attributed to the metabolic compartmentalization, which resulted in higher demand of N uptake and higher physiological use of N under sub-optimal and supra-optimal N growth conditions^24^. The shift in the slope of Nmax and Nmin curves on different bases indicated that Indica rice had higher N uptake demand, except for SDM basis, because Japonica rice had a higher proportion of structural N than that of Indica rice. These results were in consensus with previous reports on rice^27^. The slopes of Nmax curves on PDM, LAI and LDM bases decreased gradually during vegetative growth in both the rice ecotypes (Fig. 1a and b), while the slopes of the curves overlapped with each other during mid-vegetative growth on SDM basis (Fig. 1d). This overlapping was attributed to the ability of rice plant to transfer photosynthetic products from structural compartment to metabolic compartment for grain formation^28^. The gradual decline in slopes further confirmed the supra-optimal N growth conditions. The slopes of Nmin curves on different bases at early growth stages showed a similar decline in two rice groups, which became obvious towards advancing maturity (Fig. 1). The higher slope of Indica rice indicated its potential to thrive better than Japonica rice under adverse conditions^24^. The initial similar slopes were attributed to lower availability of mineral N in the soil during early growth stages. The overlapping of Nmin curves on LAI basis (Fig. 1b) in the two rice ecotypes towards advancing maturity was associated with the reduction in LA expansion and leaf senescence under sup-optimal N growth conditions.

### Comparison of N nutrition index, accumulated N deficit and N requirement from N dilution curves with different bases

The NNI, AND and NR are theoretically sound and agronomically relevant N diagnostic tools to quantify the N status of the crop based on robust N dilution curves. The increase in NNI at HD stage under optimal and supra-optimal N supply in the two rice groups, as well as on PDM, LAI and LDM bases under sub-optimal N supply in Indica rice was attributed to the ability of rice plant to translocate the photosynthates from vegetative to reproductive organs for grain formation^29^. The decrease in NNI on SDM basis (Fig. 2h) in contrast to PDM, LAI and LDM based NNI of Indica rice under sub-optimal N supply were associated with the superior ability of Indica rice to transfer photosynthetic products from structural to metabolic component^28^.

The almost similar values of AND and NR on PDM and LAI basis in two rice ecotypes (Figs 3 and 4a–d), except at HD stage, indicated that rate of PDM accumulation and LA expansion were similar during vegetative growth, yet there was an over-estimation of AND and NR in case of the N0 treatment, where N fertilizer was not applied. A similar trend in cereals^30^ was attributed to greater N supply from indigenous soil resources and higher N use efficiency under N0 treatment. The differences in AND and NR on LAI basis against other approaches in both the rice ecotypes at HD stage were attributed to senescence of leaves that resulted in a reduction of LA expansion at HD stage. Moreover, the reduction in AND and NR at HD stage on LDM basis in Indica rice indicated that rate of leaf senescence was higher in Indica rice than in Japonica rice, which in turn resulted in a reduction of AND and NR at HD on PDM basis in Indica rice by changing the leaf/shoot ratio. The AND and NR exhibited significant differences in the two rice ecotypes on PDM, LDM and SDM bases, but non-significant differences in case of LAI basis, indicating that LA expansion was uniform in both the rice ecotypes under sub-optimal, optimal and supra-optimal N supplies^31^. On the other hand, Indica rice exhibited more N uptake capacity than Japonica rice. The difference of leaf/stem ratio among the two rice ecotypes was attributed to different N uptakes, which resulted in a differences between AND and NR, because AND and NR estimated from the LDM based curve were higher in Indica rice than those in Japonica rice.

The estimation of NNI, AND and NR on different plant bases is imperative, because plant growth has to be considered as the sum of metabolic and structural compartments, having their own N demand for metabolic and structural processes as well as to offer assistance for a better understanding of the concept of N dilution in crops^18^. The results of the present study indicated that the leaf based approaches (LAI and LDM), especially LDM based N parameters, might be the most appropriate substitute for PDM based N parameters, rather than that on SDM basis. Moreover, the real-time, rapid and non-destructive field methods used in modern agriculture such as chlorophyll meter, hyper-spectral meter, remote sensing and digital photography, generally monitor N concentration at a single leaf or on canopy basis, instead of entire plant basis^12^. Therefore, the in-season estimation of N parameters on LAI or LDM (leaf basis) using non-destructive tools will lead to judicious N diagnosis and management in crop production.

### Implication for N diagnosis and crop modeling

Quantification of N in an agroecosystem is imperative to diminish environmental influences and to increase crop production. N dilution curve offers information on the N status of agronomic crops accuratly and quantitatively^32^. An accurate appraisal of crop N status for developing a fertilizer decision support method requires reflection of crop N status at most critical crop growth stages^33^. The main application of the N dilution curve in crop production is to evaluate in-season crop N status. The implication of this concept as an investigative tool to make corrective decisions of N dressing recommendation during crop production can be used for a priori analysis intended to improve N management, and for a posteriori diagnosis intended to identify sub-optimal N nutrition for fields in production^6^. Based on this perception, various diagnostic tools with the purpose of improving N management were established to assess the N status of agronomic crops. The present study exhibited that the N dilution curve and resulting N parameters (NNI, AND and NR) effectively distinguished among sub-optimal, optimal and supra-optimal N nutrition conditions. These N parameters being crop specific, precise and biologically sound in relation to actual crop growth can be exploited for in-season estimation of crop N status, and to make corrective decisions for supplemental N application during the growth period of the rice crop. Moreover, these N parameters can be utilized for in-season estimation of crop growth rate, grain yield and quantification of time-course NR for decision support in production management as well as for setting yield targets according to different N application rates^1^,^5^. Additionally, it can be used in breeding programs as a phenotyping tool for selecting superior lines at HD stage, and to avoid the issues related to sub-optimal and supra-optimal N nutrition. The algorithms proposed in the present study can also be integrated with chlorophyll meter readings^16^,^32^,^34^ as well as with crop growth and management models to forecast crop N status and to quantify N dressing plan in different ecotypes of rice^1^,^5^,^16^.

These algorithms can be used to govern N accumulation in plant tissues under sub-optimal, optimal and supra-optimal N nutrition conditions. The algorithms and parameters developed in this study can be integrated in different rice crop growth models, such as RiceGrow, WARM, ORYZA2000, APSIM-ORYZA, and CERES-Rice^35^,^36^,^37^,^38^,^39^ for their application under different climatic conditions, genotype and management practices, in which their N diagnosis methods are not valid. The findings of this study imply that a re-analysis of the N and PDM relations to investigate the N dependency on crop growth stages and determination of new N dilution curves relating plant and specific organ N concentration to LAI, LDM, and SDM at different crop stages of rice, rather than only using classical PDM approach, would offer greater benefit for improved understanding of N dilution curve. Furthermore, separately determining N dilution curve on LAI and specific organ DM basis is crucial in order to determine the genotypic and environmental differences of crop N dynamics in a physiologically functional manner for developing crop simulation models^32^. The study also raises the necessity of rigorous updates with new dataset and integration of crop growth stages and different bases for determination of new critical N dilution curves in crop species for which the curves were developed 15–20 years ago, in order to meet the increasing demand for the model to be confidently applied in research areas and geographical regions other than those where the model was developed. Use of the newly derived threshold N concentrations would lead to improvement in simulations of crop growth (DM accumulation, LA expansion) and N uptake.

For the first time, the present study made a comprehensive comparison of critical N dilution curves with different plant bases in Japonica and Indica rice. The results elucidated that a single N dilution curve developed in one ecotype of rice could be used for both ecotypes. Use of the newly derived threshold N concentrations from the N dilution curves would lead to improvements in simulations of crop growth and N uptake, as well as in N dressing management and N use efficiency, thus contribute towards sustainable rice production. Although N parameters calculated in the present study distinguished well between sub-optimal and supra-optimal N nutrition conditions, yet a more comprehensive investigations under diverse environmental conditions and production systems with different crop species would extend the validity of this approach in broader aspects.

## Methods

### Study site

The study was conducted in Jiangsu province of China, which is located in Yangtze River Reaches. The region is one of the major cultivated regions of China and contributes around 65% of the nation’s rice production. The area containing these sites is categorized by a subtropical-temperate climate with cold winter and hot summer and is appropriate for planting different ecotypes of rice. The region receives 2177 h of sunshine and 1030 mm rainfall, annually. The detailed soil characteristics and cropping practices of all sites are shown in Table 2.

### Experimental design and crop management

Eight multi-N rates (0 to 375 kg N ha^−1^) field experiments, using six rice cultivars, (three Japonica rice hybrids, including Lingxiangyou-18 (LXY-18), Wuxiangjing-14 (WXJ-14) and Wuyunjing-24 (WYJ-24), and three Indica rice hybrids Liangyoupei-9 (LYP-9), Shanyou-63 (SY-63) and Y-Liangyou-1 (YLY-1)), were carried out in the present study. A randomized complete block design was used with three replications in all eight experiments. The size of each plot was 4.6 m × 8 m in 2008 and 2009, 4.5 m × 8 m in 2010 and 2011, and 5 m × 6 m in 2012, 2013 and 2014. The inter-row spacing of 30 cm was used at all sites. The planting density was approximately 22.2 × 10^4^ plants ha^−1^ in all experiments. In all the experiment, each plot received 135 kg P_2_O_5_ ha^−1^ and 190 kg K_2_O ha^−1^ before transplanting. All experiments were carried out with suitable crop management according to each site, in order to obtain the potential yield (N fertilizer was the only limiting factor). The detailed information about N treatments in eight field experiments including N rates, N distribution (%) and N application timings are summarized in Table 3.

### Plant sampling and measurements

Plant samples (5 plants) were collected from 0.23 m^2^ area at active tillering (AT), mid tillering (MT), stem elongation (SE), panicle initiation (PI), booting (BT) and heading (HD) stages during the vegetative phase for growth analysis. The plant samples were divided into leaf (green leaf blade), stem (culm plus sheath). Green LA was measured with a leaf area meter (LI-3000, LI-COR, Lincoln, NE, USA) and expressed as LAI. All the samples were oven-dried for 30 min at 105 °C to quickly cease plant metabolic activities, followed by 70 °C to constant weight to attain the PDM, LDM, and SDM (tha^−1^). After oven drying to a constant weight, each component was subsequently ground to powder for analytical N determination. Samples of 0.2 g dried and ground samples were digested using a mixture of H_2_O_2_ and H_2_SO_4_, and the N content was determined using a continuous-flow auto-analyzer (BRAN + LUEBBE AA3; Germany)^34^. The N accumulation (kg N ha^−1^) was calculated by multiplying plant DM. LAI, LDM and SDM by N concentration of the respective plant tissue^9^.

### Statistical analysis

The data for determination of critical N points were analyzed according to the methodology proposed by Justes *et al*.^6^. For each sampling date, year and rice ecotypes, the amounts of PDM, LDM, SDM and LAI produced with the varied N rates and the corresponding tissue N concentrations were subjected to analysis of variance (ANOVA) using GLM procedures in IBM SPSS Version19.0 (IBM Corporation, Armonk, New York). The differences between treatment means were assessed using least significant difference (LSD) test at 90% level of significance, instead of classically using 95% in order to reduce the occurrence of Type II errors^1^,^5^,^16^. The variation in the tissue N concentration versus PDM, LAI, LDM and SDM across the different N levels was combined into a bilinear relation composed of a linear regression representing the joint increase in tissue N concentration and PDM, LAI, LDM and SDM and a vertical line corresponding to an increase in tissue N concentration without significant variation in PDM, LAI, LDM and SDM. The theoretical Nc points corresponds to the ordinate of the breakout of the bilinear regression. The bilinear regression between PDM, LDM, SDM and LAI and tissue N concentration (%) was conducted using Microsoft Excel 2010 (Microsoft Corporation, Redmond, WA, USA).

### Construction of critical N dilution curves

The critical N dilution curves were determined according to the methodology proposed by Justes *et al*.^6^. The data points from eight field experiments were identified, for which N did not limit growth (supra-optimal N nutrition) or was not in excess (sub-optimal N nutrition). The sub-optimal N treatment was defined as a treatment for which a supplement of N application led to a significant increase in PDM, LDM, SDM and LAI. The supra-optimal N treatment is defined as a treatment for which a supplement of N application did not lead to an increase in PDM, LDM, SDM and LAI and, at the same time, included a significant increase in tissue N concentration. If at the same measurement date, statistical analysis distinguished at least one set of sub-optimal N nutrition and supra-optimal N nutrition data points, this series of data could be used to define the N dilution curve. An allometric function based on power regression model (Freundlich model) was used to determine the relationship between the observed decreases in N concentration with increasing PDM, LDM, SDM, and LAI.

### Maximum and minimum nitrogen curves

The maximum N dilution curves (Nmax) and minimum N dilution curves (Nmin) were developed to set the upper and lower limit curves. The Nmax (a curve corresponding to the maximum N uptake), was obtained with increasing N treatments for maximum growth and N accumulation rates. The Nmin is considered as a lower limit at which the N metabolism would soon stop to function. It corresponds to the minimum N taken up by rice plants in present study. The data points from the most plethoric N treatments were supposed to represent the Nmax, whereas the Nmin was determined using the data points from the most sub-optimal treatments for which N application was zero (N0 check plots)^40^.

#### Nitrogen nutrition index

The NNI, the ratio between the actual crop N concentration (Na) and critical N concentration (Nc), of different rice cultivars at different vegetative growth stages was calculated from Justes *et al*.^6^:


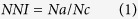


where Na and Nc represented actual N concentration and critical N concentration of tissue, respectively If NNI = 1, N nutrition were considered as optimum, while NNI >1 and NNI <1 indicated excess and deficient N nutrition, respectively.

#### Accumulated nitrogen deficit

The AND, is the amount of N, which a crop fails to take up at any stage of development for reaching the level of critical N uptake (i.e. the deficit of crop N uptake which is required to achieve maximum yield) of different cultivars/ecotypes of rice at different growth stages was calculated according to the method proposed by Ata-Ul-Karim *et al*.^9^:


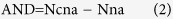


where Ncna is the N accumulation under the Nc growth condition, and Nna is the actual N accumulation under different N rates. Ncna can be obtained by multiplying the critical PDM, LAI, LDM, and SDM with crossponding critical N concentration at each growth stage. If AND = 0, N nutrition was considered as optimal, while AND >0 or AND <0 indicated excess and deficient N nutrition, respectively.

#### Nitrogen requirement

Nitrogen requirement (kg N ha^−1^), the N fertilizer requirement for a crop at any stage of development for reaching the Nc level (i.e. the crop N status corresponding to maximum growth) of rice cultivars/ecotypes at different growth stages was calculated as Ata-Ul-Karim *et al*.^1^,^5^:


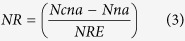


where NRE is the N recovery use efficiency of in-season N fertilizer application.

The NRE, the ratio of plant N to N supply at various crop growth was calculated for different rice cultivars/ecotypes, years, and sites from Novoa and Loomis^26^:


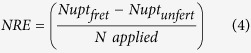


where Nupt_fert_ is N uptake in the fertilized plot and Nupt_unfert_ is the N uptake in the corresponding unfertilized plot. The NR = 0 indicated an optimum N supply, while NR >zero or the NR < zero represented the N deficit and luxury N uptake, respectively.

The N uptake in the fertilized and unfertilized plot, as well as N applied, was calculated according to the N distribution at each growth stage and year to calculate NRE at each growth stage.

### Relationship between plant based and leaf area index/organ based N parameters

The linear regression relationships between NNI, AND and NR on PDM basis and those based on LAI, LDM and SDM in three rice groups at different growth stages during vegetative period were established using Microsoft Excel 2010 (Microsoft Corporation, Redmond, WA, USA). The NNI, AND and NR values were calculated for each experiment, and then averaged for the three rice groups separately. To avoid the over or under estimation of NNI, AND and NR, the N treatments in different experiments were regrouped as N0 (no N application), N1 (75, 80 and 90 kg N ha^−1^), N2 (150, 160 and 180 kg N ha^−1^), N3 (225 and 240 kg N ha^−1^), N4 (270, 300 and 320 kg N ha^−1^) and N5 (360 and 375 kg N ha^−1^).

## Additional Information

**How to cite this article:** Ata-Ul-Karim, S. T. *et al*. Comparison of different critical nitrogen dilution curves for nitrogen diagnosis in rice. *Sci. Rep.* **7**, 42679; doi: 10.1038/srep42679 (2017).

**Publisher's note:** Springer Nature remains neutral with regard to jurisdictional claims in published maps and institutional affiliations.

## Supplementary Material

Supplementary Information

## Figures and Tables

**Table 1:** Values of the coefficients *a* and *b* for the existing nitrogen dilution curves in Japonica and Indica rice used for comparison.

Ecotype	Climate	Plant index	Individual curve		Unified curve		References
			*a*	*b*	*a*	*b*	
**Japonica**	**Subtropical**	PDM	3.41/3.94	0.30	3.53	0.28	(9)
		LAI	3.57/4.03	0.37	3.70	0.35	(10)
		LDM	3.58/4.11	0.25	3.76	0.22	(12)
		SDM	2.08/2.33	0.29	2.17	0.27	(14)
**Indica**	**Tropical**	PDM	5.35/4.194.48/5.09	0.50/0.390.52/0.52	5.2	0.50	(8)
		LAI			3.22	0.16	(17)

PDM, plant dry matter, LAI, leaf area index, LDM, leaf dry matter and SDM, stem dry matter.

**Table 2:** Site characteristics and cropping practices for the eight field experiments.

Soil and crop information	Experiment 1 (2008)	Experiment 2 (2009)	Experiment 3 (2010)	Experiment 4 (2011)	Experiment 5 (2011)	Experiment 6 (2012)	Experiment 7 (2013)	Experiment 8 (2014)
	Jiangning (31°56' N, 118°59' E)		Yizheng (32°16' N, 119°10' E)			Rugao (32°23' N, 120°33' E)		
**Soil type**	Ultisols	Ultisols	Ultisols	Ultisols	Ultisols	Ultisols	Ultisols	Ultisols
**Soil pH**	6.3	6.4	6.2	6.4	6	6	6.1	6.4
**Organic matter**	18.1 g kg^−1^	15.5 g kg^−1^	17.5 g kg^−1^	15.5 g kg^−1^	19.2 g kg^−1^	13.5 g kg^−1^	14.9 g kg^−1^	23 g kg^−1^
**Total N**	1.28 g kg^−1^	1.3 g kg^−1^	1.6 g kg^−1^	1.3 g kg^−1^	1.7 g kg^−1^	1.5 g kg^−1^	1.1 g kg^−1^	1.35 g kg^−1^
**Available P**	27.6 mg g^−1^	32 mg g^−1^	43 mg g^−1^	38 mg g^−1^	42 mg g^−1^	30 mg g^−1^	32 mg g^−1^	46.2 mg g^−1^
**Available K**	75.2 mg g^−1^	80 mg g^−1^	90 mg g^−1^	85 mg g^−1^	95 mg g^−1^	84 mg g^−1^	80 mg g^−1^	105.5 mg g^−1^
**Previous crop**	Wheat	Wheat	Wheat	Wheat	Wheat	Wheat	Wheat	Wheat
**Planting date**	20-Jun	17-Jun	21-Jun	21-Jun	18-Jun	20-Jun	20-Jun	21-Jun
**Harvesting date**	24-Oct	24-Oct	16-Oct	16-Oct	18-Oct	24-Oct	24-Oct	16-Oct
**Cultivars**	LYP-9	LYP-9	LXY-18, WXJ-14	LXY-18, WXJ-14	SY-63	SY-63	WXJ-14, SY-63	WYJ-24, YLY-1
**Ecotype**	Indica	Indica	Japonica	Japonica	Indica	Indica	Japonica, Indica	Japonica, Indica

**Table 3:** Nitrogen application and sampling details for the eight field experiments.

Experiment No.	N rate (kg ha^−1^)	N distribution (%)	N application timing	Sampling stage	Sampling date
**Experiment 1 (2008)**	N0 (0),	50%	PP,	MT,	15-Jul
	N1 (110),	10%	AT,	SE,	25-Jul
	N2 (220),	20%	PI,	PI,	5-Aug
	N3 (330)	20%	BT	BT,	17-Aug
				HD	31-Aug
**Experiment 2 (2009)**	N0 (0),	50%	PP,	MT,	16-Jul
	N1 (110),	10%	AT,	SE,	26-Jul
	N2 (220),	20%	PI,	PI,	7-Aug
	N3 (330)	20%	BT	BT,	22-Aug
				HD	1-Sep
**Experiment 3 (2010)**	N0 (0),	50%	PP,	AT,	7-Jul
	N1 (80),	10%	AT,	MT,	17-Jul
	N2 (160),	20%	PI,	SE,	26-Jul
	N3 (240)	20%	BT	PI,	8-Aug
	N4 (320)			BT,	20-Aug
				HD	30-Aug
**Experiment 4 (2011)**	N0 (0),	50%	PP,	AT,	9-Jul
	N1 (90),	10%	AT,	MT,	21-Jul
	N2 (180),	20%	PI,	SE,	26-Jul
	N3 (270),	20%	BT	PI,	2-Aug
	N4 (360)			BT,	25-Aug
				HD	3-Sep
**Experiment 5 (2011)**	N0 (0),	50%	PP,	AT,	10-Jul
	N1 (70),	10%	AT,	MT,	21-Jul
	N2 (170),	20%	PI,	SE,	30-Jul
	N3 (270),	20%	BT	PI,	9-Aug
	N4 (370)			BT,	18-Aug
				HD	30-Aug
**Experiment 6 (2012)**	N0 (0),	50%	PP,	AT,	9-Jul
	N1 (70),	10%	AT,	MT,	20-Jul
	N2 (170),	20%	PI,	SE,	30-Jul
	N3 (270),	20%	BT	PI,	9-Aug
	N4 (370)			BT,	19-Aug
				HD	29-Aug
**Experiment 7 (2013)**	N0 (0),	40%	PP,	AT,	9-Jul
	N1 (75),	10%	AT,	MT,	19-Jul
	N2 (150),	30%	PI,	SE,	29-Jul
	N3 (225),	20%	BT	PI,	9-Aug
	N4 (300),			BT,	19-Aug
	N5 (375)			HD	28-Aug
**Experiment 8 (2014)**	N0 (0),	40%	PP,	AT,	5-Jul
	N1 (150),	10%	AT,	MT,	18-Jul
	N2 (225),	30%	PI,	SE,	27-Jul
	N3 (300),	20%	BT	PI,	6-Aug
	N4 (375)			BT,	16-Aug
				HD	26-Aug

PP, pre-planting, AT, active tillering, MT, mid tillering, SE, stem elongation, PI, panicle initiation, BT, booting and HD, heading.
