# Supplementary material for: Comparison of different critical nitrogen dilution curves for nitrogen diagnosis in rice
Source: Sci Rep. 2017 Mar 6;7:42679. doi: 10.1038/srep42679 (PMC5338017; doi:10.1038/srep42679)
Supplement: Supplementary Information [file srep42679-s1.pdf]

Comparison of different critical nitrogen dilution curves for nitrogen diagnosis in rice

Names of authors: Syed Tahir Ata-Ul-Karim, Yan Zhu, Xiaojun Liu, Qiang Cao, Yongchao Tian, Weixing Cao*

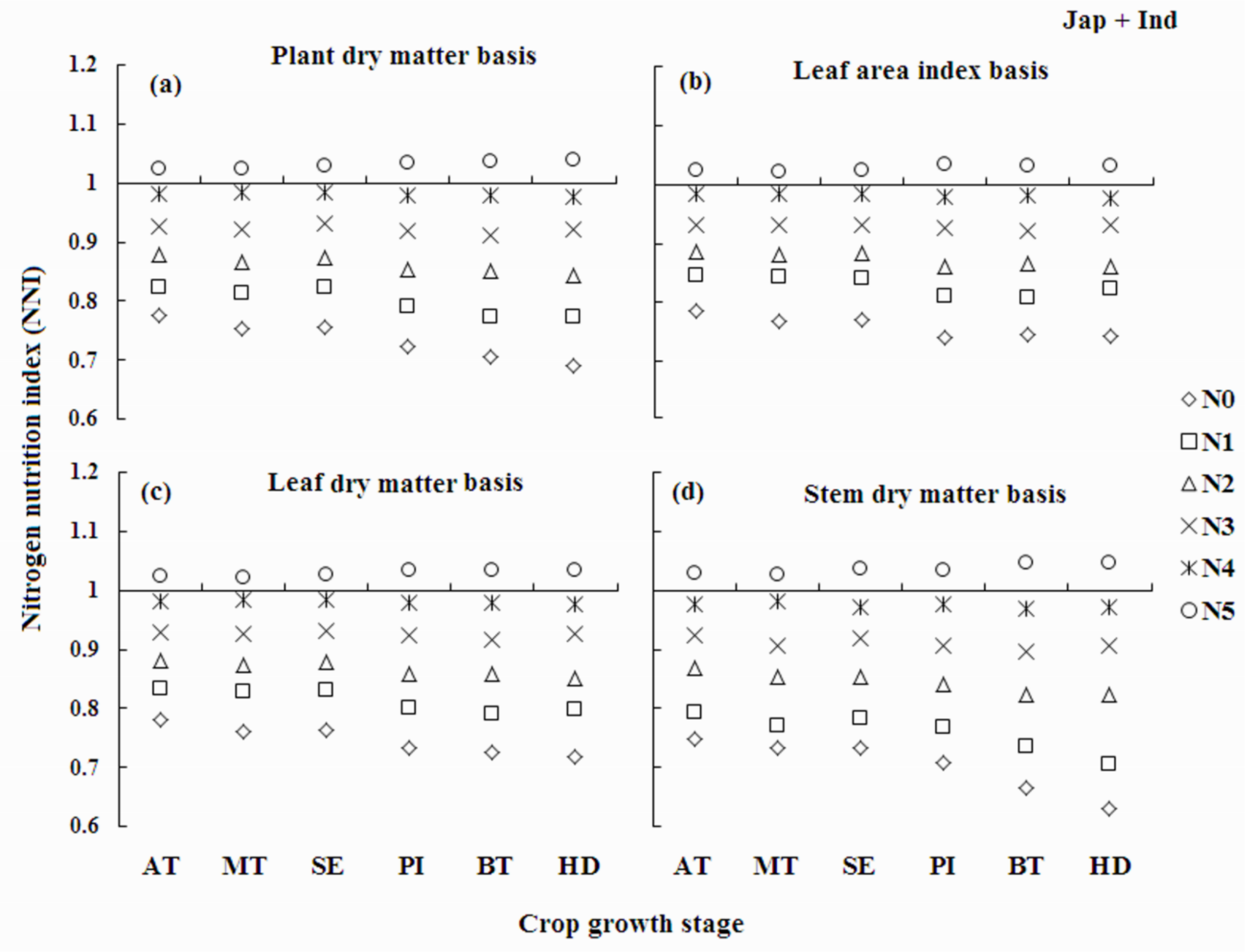

Supplementary Fig. 1 Nitrogen nutrition index (NNI) of Japonica+Indica rice at different growth stages in eight varied N rates experiments on the bases of different N dilution curves (a: plant dry matter basis; b: leaf area index basis; c: leaf dry matter basis; d: stem dry matter basis). For X-axis, AT represents active tillering, MT mid tillering, SE stem elongation, PI panicle initiation, BT booting, and HD heading.

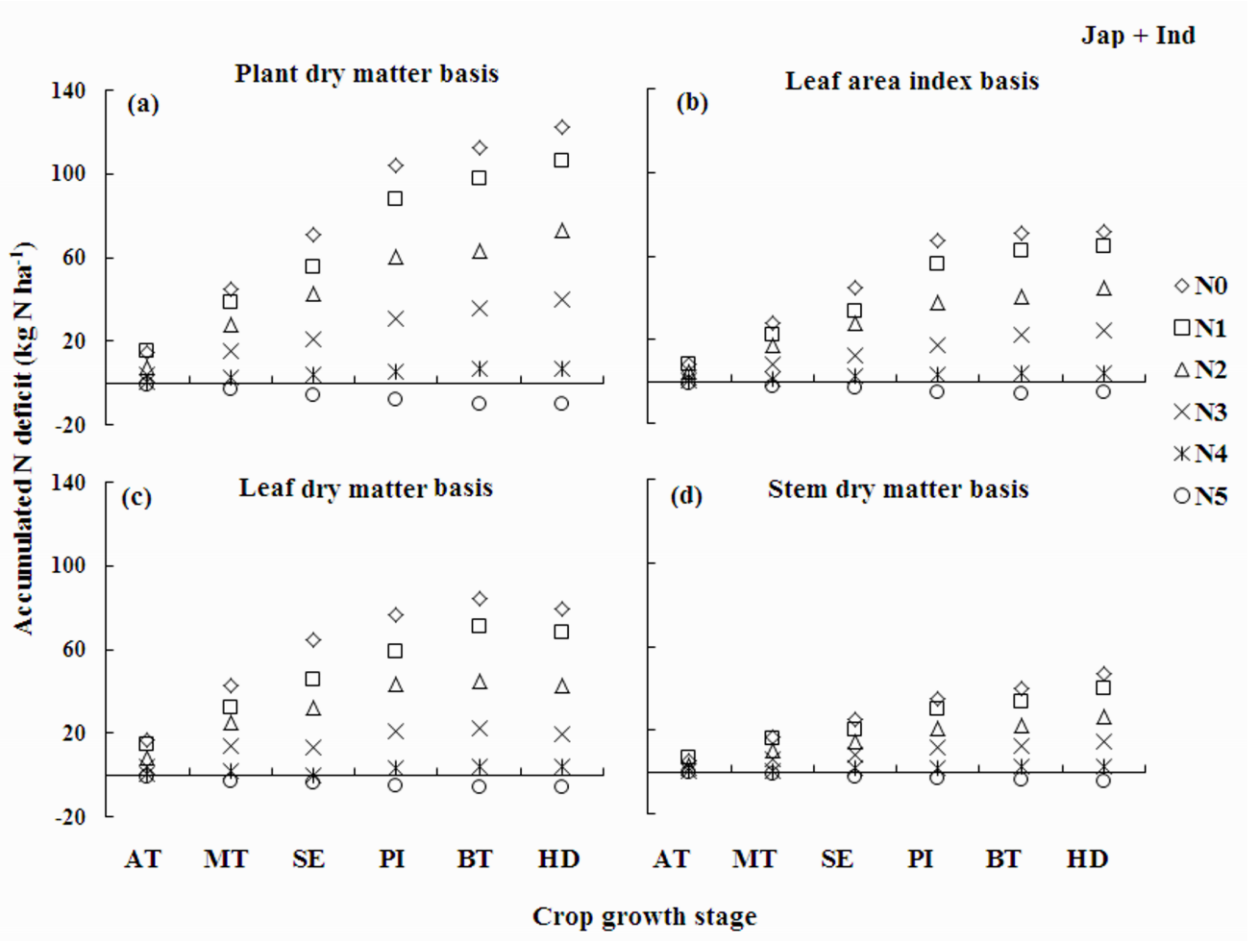

Supplementary Fig. 2 Accumulated nitrogen deficit (AND, kg ha^{-1}) of Japonica+Indica rice at different growth stages in eight varied N rates experiments on the bases of different N dilution curves (a: plant dry matter basis; b: leaf area index basis; c: leaf dry matter basis; d: stem dry matter basis). For X-axis, AT represents active tillering, MT mid tillering, SE stem elongation, PI panicle initiation, BT booting, and HD heading.

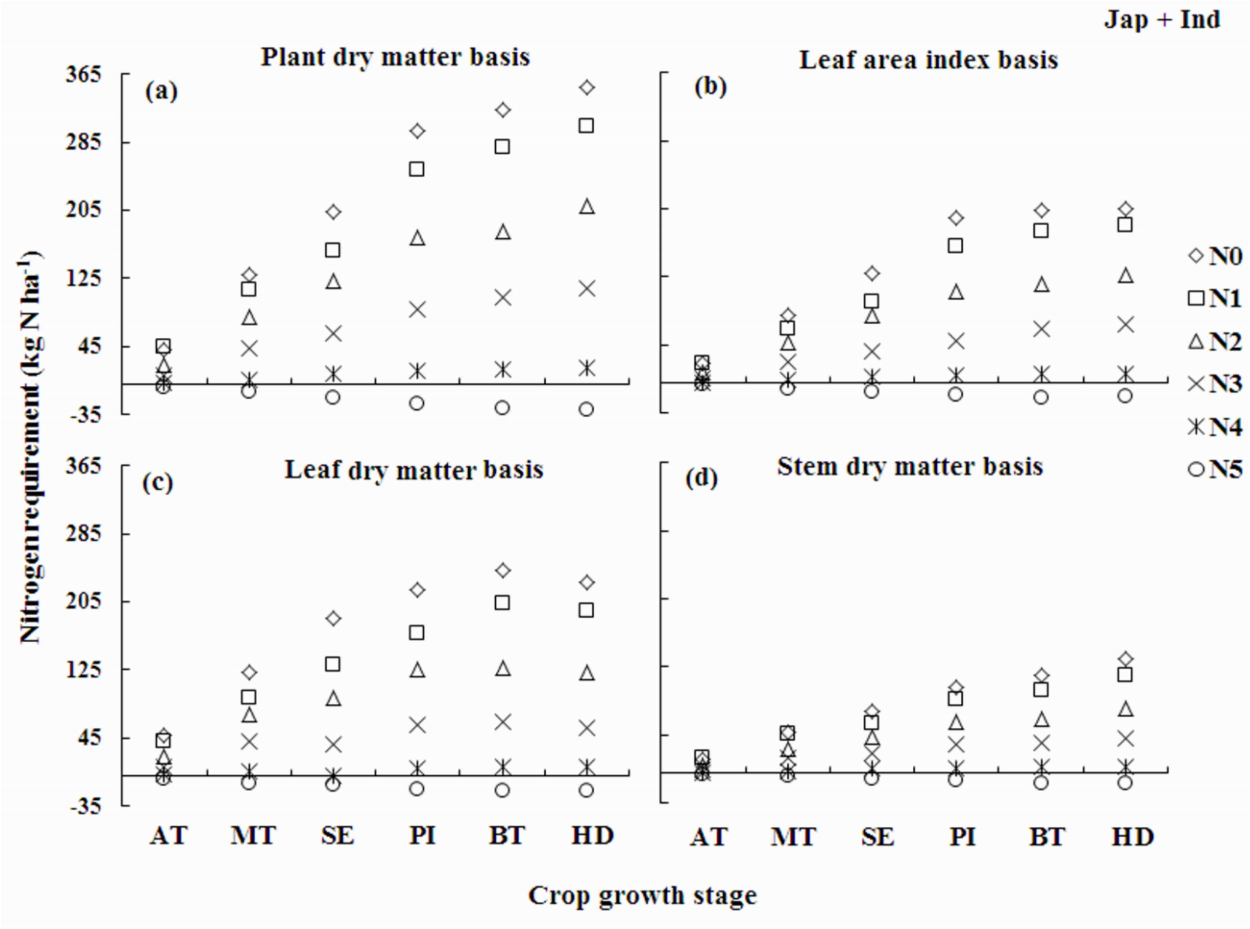

Supplementary Fig. 3 Nitrogen requirement (NR, kg ha^{-1}) of Japonica+Indica rice at different growth stages in eight varied N rates experiments on the bases of different N dilution curves (a: plant dry matter basis; b: leaf area index basis; c: leaf dry matter basis; d: stem dry matter basis). For X-axis, AT represents active tillering, MT mid tillering, SE stem elongation, PI panicle initiation, BT booting, and HD heading.

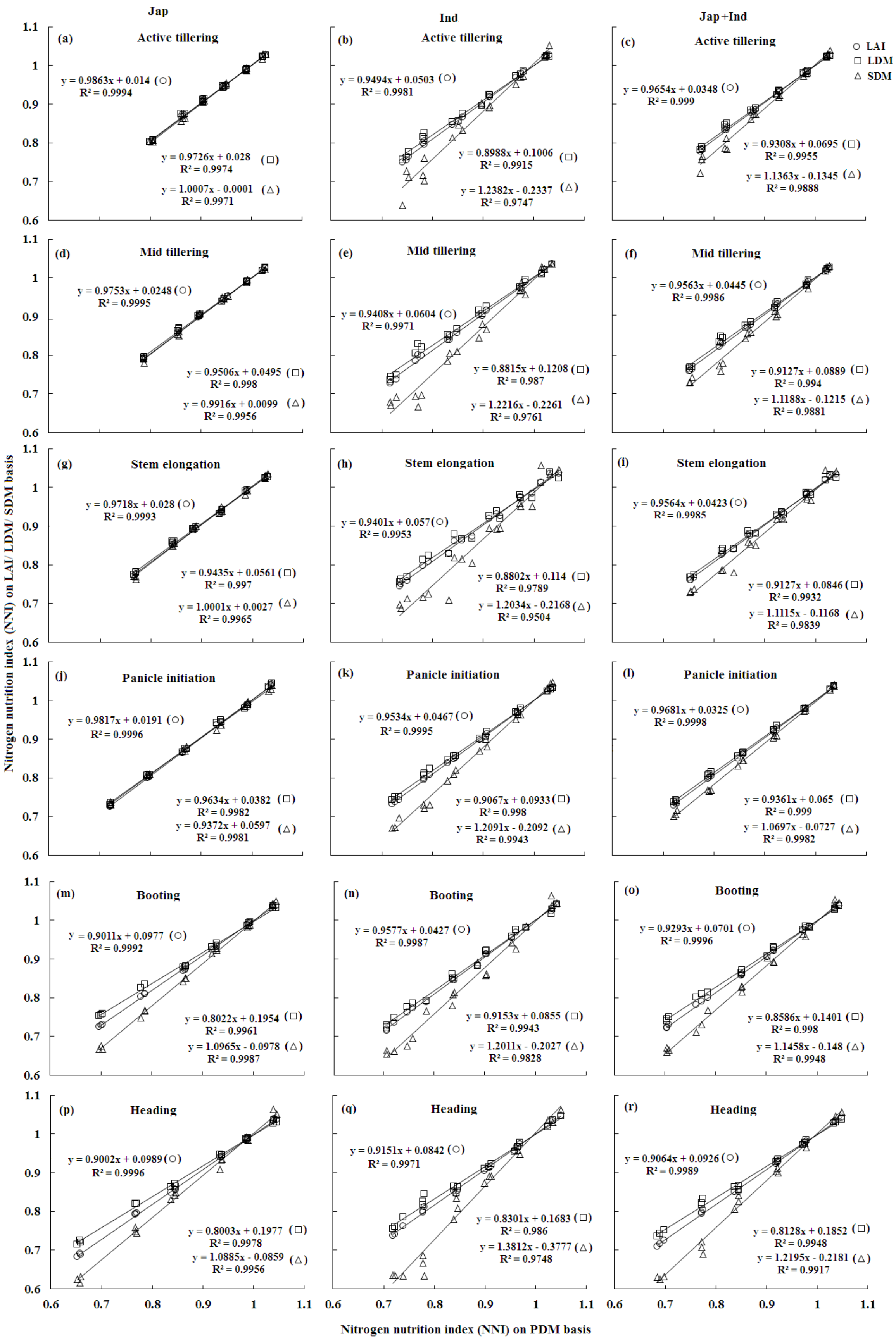

Jap
Ind
Jap+Ind
LAI
LDM
SDM
(a) Active tillering
y = 0.9863x + 0.014 (○)
R² = 0.9994
y = 0.9726x + 0.028 (□)
R² = 0.9974
y = 1.0007x - 0.0001 (△)
R² = 0.9971
(b) Active tillering
y = 0.9494x + 0.0503 (○)
R² = 0.9981
y = 0.8988x + 0.1006 (□)
R² = 0.9915
y = 1.2382x - 0.2337 (△)
R² = 0.9747
(c) Active tillering
y = 0.9654x + 0.0348 (○)
R² = 0.999
y = 0.9308x + 0.0695 (□)
R² = 0.9955
y = 1.1363x - 0.1345 (△)
R² = 0.9888
(d) Mid tillering
y = 0.9753x + 0.0248 (○)
R² = 0.9995
y = 0.9506x + 0.0495 (□)
R² = 0.998
y = 0.9916x + 0.0099 (△)
R² = 0.9956
(e) Mid tillering
y = 0.9408x + 0.0604 (○)
R² = 0.9971
y = 0.8815x + 0.1208 (□)
R² = 0.987
y = 1.2216x - 0.2261 (△)
R² = 0.9761
(f) Mid tillering
y = 0.9563x + 0.0445 (○)
R² = 0.9986
y = 0.9127x + 0.0889 (□)
R² = 0.994
y = 1.1188x - 0.1215 (△)
R² = 0.9881
(g) Stem elongation
y = 0.9718x + 0.028 (○)
R² = 0.9993
y = 0.9435x + 0.0561 (□)
R² = 0.997
y = 1.0001x + 0.0027 (△)
R² = 0.9965
(h) Stem elongation
y = 0.9401x + 0.057(○)
R² = 0.9953
y = 0.8802x + 0.114 (□)
R² = 0.9789
y = 1.2034x - 0.2168 (△)
R² = 0.9504
(i) Stem elongation
y = 0.9564x + 0.0423 (○)
R² = 0.9985
y = 0.9127x + 0.0846 (□)
R² = 0.9932
y = 1.1115x - 0.1168 (△)
R² = 0.9839
(j) Panicle initiation
y = 0.9817x + 0.0191 (○)
R² = 0.9996
y = 0.9634x + 0.0382 (□)
R² = 0.9982
y = 0.9372x + 0.0597 (△)
R² = 0.9981
(k) Panicle initiation
y = 0.9534x + 0.0467 (○)
R² = 0.9995
y = 0.9067x + 0.0933 (□)
R² = 0.998
y = 1.2091x - 0.2092 (△)
R² = 0.9943
(l) Panicle initiation
y = 0.9681x + 0.0325 (○)
R² = 0.9998
y = 0.9361x + 0.065 (□)
R² = 0.999
y = 1.0697x - 0.0727 (△)
R² = 0.9982
(m) Booting
y = 0.9011x + 0.0977 (○)
R² = 0.9992
y = 0.8022x + 0.1954 (□)
R² = 0.9961
y = 1.0965x - 0.0978 (△)
R² = 0.9987
(n) Booting
y = 0.9577x + 0.0427 (○)
R² = 0.9987
y = 0.9153x + 0.0855 (□)
R² = 0.9943
y = 1.2011x - 0.2027 (△)
R² = 0.9828
(o) Booting
y = 0.9293x + 0.0701 (○)
R² = 0.9996
y = 0.8586x + 0.1401 (□)
R² = 0.998
y = 1.1458x - 0.148 (△)
R² = 0.9948
(p) Heading
y = 0.9002x + 0.0989 (○)
R² = 0.9996
y = 0.8003x + 0.1977 (□)
R² = 0.9978
y = 1.0885x - 0.0859 (△)
R² = 0.9956
(q) Heading
y = 0.9151x + 0.0842 (○)
R² = 0.9971
y = 0.8301x + 0.1683 (□)
R² = 0.986
y = 1.3812x - 0.3777 (△)
R² = 0.9748
(r) Heading
y = 0.9064x + 0.0926 (○)
R² = 0.9989
y = 0.8128x + 0.1852 (□)
R² = 0.9948
y = 1.2195x - 0.2181 (△)
R² = 0.9917
Nitrogen nutrition index (NNI) on LAI/ LDM/ SDM basis
Nitrogen nutrition index (NNI) on PDM basis
0.6
0.7
0.8
0.9
1
1.1

Supplementary Fig. 4 The relationships between plant dry matter based nitrogen nutrition index (NNI) and leaf area index, leaf dry matter and stem dry matter based NNI at different growth stages (a, b, c: active tillering; d, e, f: mid tillering; g, h, i: stem elongation; j, k, l: panicle initiation; m, n, o: booting; p, q, r: heading stages) for Japonica, Indica and Japonica+ Indica rice, respectively.

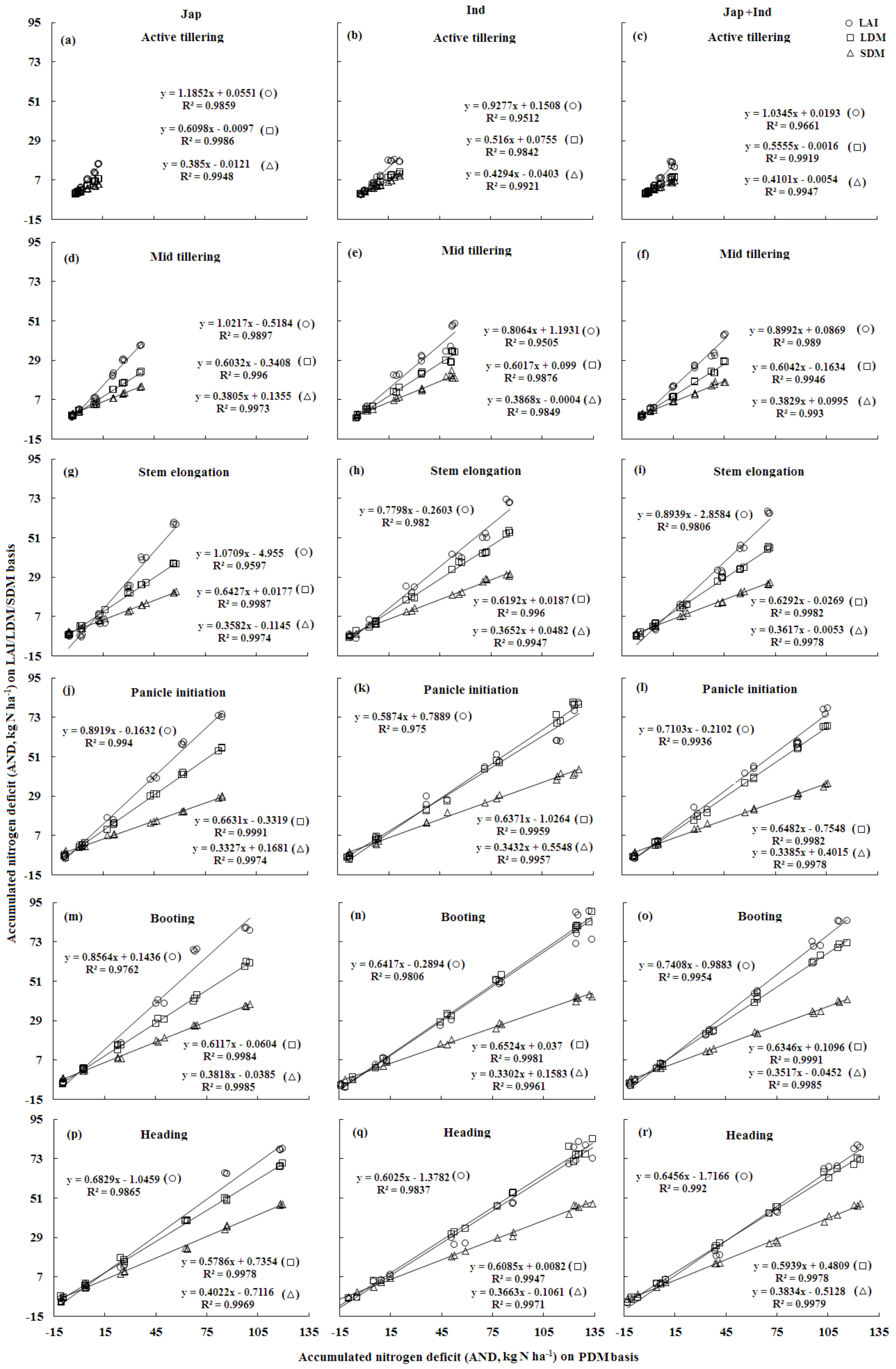

Jap
Ind
Jap+Ind
○ LAI
□ LDM
△ SDM
(a) Active tillering
y = 1.1852x + 0.0551 (○)
R² = 0.9859
y = 0.6098x - 0.0097 (□)
R² = 0.9986
y = 0.385x - 0.0121 (△)
R² = 0.9948
(b) Active tillering
y = 0.9277x + 0.1508 (○)
R² = 0.9512
y = 0.516x + 0.0755 (□)
R² = 0.9842
y = 0.4294x - 0.0403 (△)
R² = 0.9921
(c) Active tillering
y = 1.0345x + 0.0193 (○)
R² = 0.9661
y = 0.5555x - 0.0016 (□)
R² = 0.9919
y = 0.4101x - 0.0054 (△)
R² = 0.9947
(d) Mid tillering
y = 1.0217x - 0.5184 (○)
R² = 0.9897
y = 0.6032x - 0.3408 (□)
R² = 0.996
y = 0.3805x + 0.1355 (△)
R² = 0.9973
(e) Mid tillering
y = 0.8064x + 1.1931 (○)
R² = 0.9505
y = 0.6017x + 0.099 (□)
R² = 0.9876
y = 0.3868x - 0.0004 (△)
R² = 0.9849
(f) Mid tillering
y = 0.8992x + 0.0869 (○)
R² = 0.989
y = 0.6042x - 0.1634 (□)
R² = 0.9946
y = 0.3829x + 0.0995 (△)
R² = 0.993
(g) Stem elongation
y = 1.0709x - 4.955 (○)
R² = 0.9597
y = 0.6427x + 0.0177 (□)
R² = 0.9987
y = 0.3582x - 0.1145 (△)
R² = 0.9974
(h) Stem elongation
y = 0.7798x - 0.2603 (○)
R² = 0.982
y = 0.6192x + 0.0187 (□)
R² = 0.996
y = 0.3652x + 0.0482 (△)
R² = 0.9947
(i) Stem elongation
y = 0.8939x - 2.8584 (○)
R² = 0.9806
y = 0.6292x - 0.0269 (□)
R² = 0.9982
y = 0.3617x - 0.0053 (△)
R² = 0.9978
(j) Panicle initiation
y = 0.8919x - 0.1632 (○)
R² = 0.994
y = 0.6631x - 0.3319 (□)
R² = 0.9991
y = 0.3327x + 0.1681 (△)
R² = 0.9974
(k) Panicle initiation
y = 0.5874x + 0.7889 (○)
R² = 0.975
y = 0.6371x - 1.0264 (□)
R² = 0.9959
y = 0.3432x + 0.5548 (△)
R² = 0.9957
(l) Panicle initiation
y = 0.7103x - 0.2102 (○)
R² = 0.9936
y = 0.6482x - 0.7548 (□)
R² = 0.9982
y = 0.3385x + 0.4015 (△)
R² = 0.9978
(m) Booting
y = 0.8564x + 0.1436 (○)
R² = 0.9762
y = 0.6117x - 0.0604 (□)
R² = 0.9984
y = 0.3818x - 0.0385 (△)
R² = 0.9985
(n) Booting
y = 0.6417x - 0.2894 (○)
R² = 0.9806
y = 0.6524x + 0.037 (□)
R² = 0.9981
y = 0.3302x + 0.1583 (△)
R² = 0.9961
(o) Booting
y = 0.7408x - 0.9883 (○)
R² = 0.9954
y = 0.6346x + 0.1096 (□)
R² = 0.9991
y = 0.3517x - 0.0452 (△)
R² = 0.9985
(p) Heading
y = 0.6829x - 1.0459 (○)
R² = 0.9865
y = 0.5786x + 0.7354 (□)
R² = 0.9978
y = 0.4022x - 0.7116 (△)
R² = 0.9969
(q) Heading
y = 0.6025x - 1.3782 (○)
R² = 0.9837
y = 0.6085x + 0.0082 (□)
R² = 0.9947
y = 0.3663x - 0.1061 (△)
R² = 0.9971
(r) Heading
y = 0.6456x - 1.7166 (○)
R² = 0.992
y = 0.5939x + 0.4809 (□)
R² = 0.9978
y = 0.3834x - 0.5128 (△)
R² = 0.9979
95
73
51
29
7
-15
-15
15
45
75
105
135
Accumulated nitrogen deficit (AND, kg N ha⁻¹) on LAI/LDM/SDM basis
Accumulated nitrogen deficit (AND, kg N ha⁻¹) on PDM basis

Supplementary Fig. 5 The relationships between plant dry matter based accumulated nitrogen deficit (AND, kg ha^{-1}) and leaf area index, leaf dry matter and stem dry matter based NR at different growth stages (a, b, c: active tillering; d, e, f: mid tillering; g, h, i: stem elongation; j, k, l: panicle initiation; m, n, o: booting; p, q, r: heading stages) for Japonica, Indica and Japonica+Indica rice, respectively.

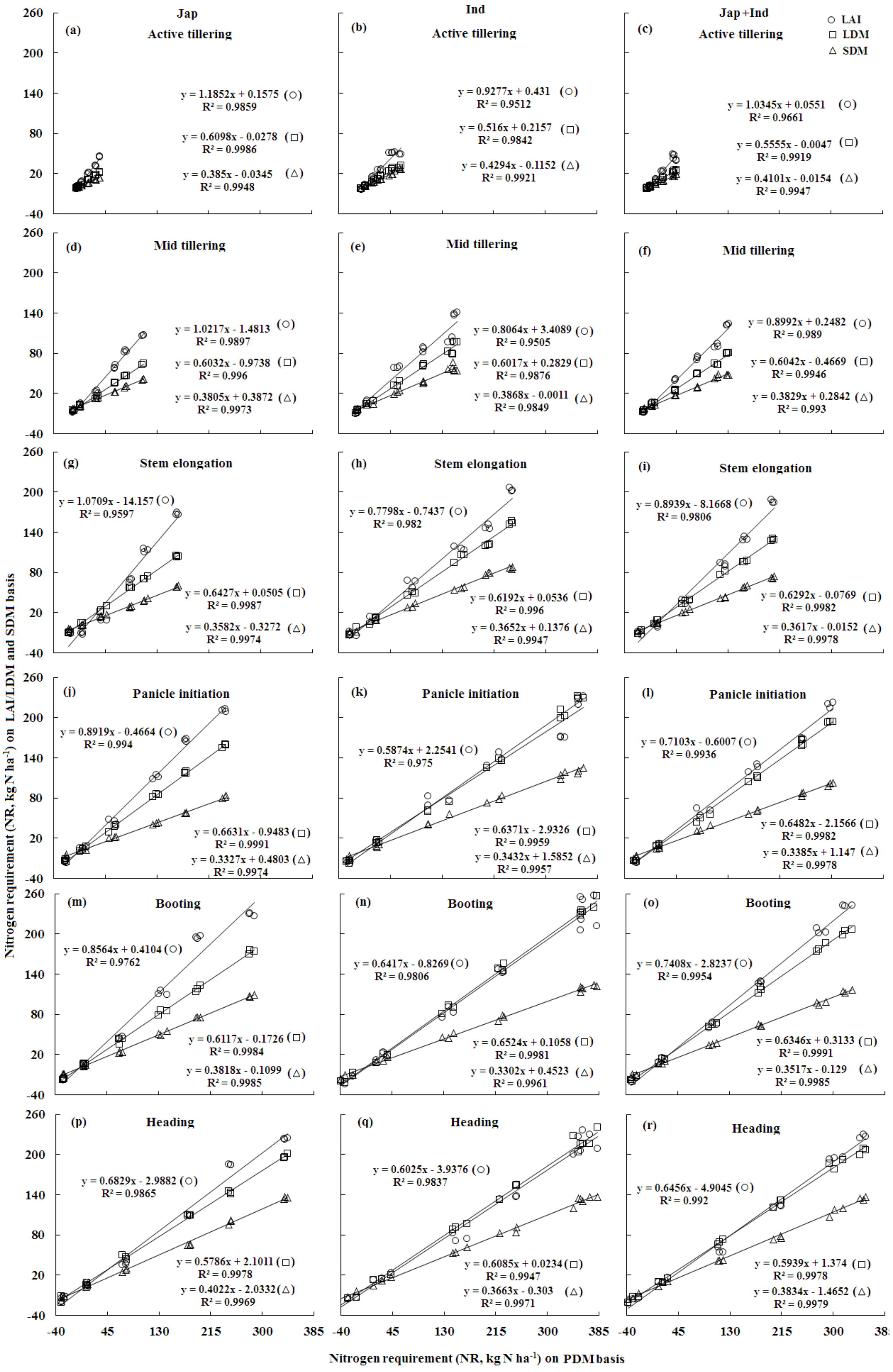
Jap
Ind
Jap+Ind
LAI
LDM
SDM
(a) Active tillering
y = 1.1852x + 0.1575 (○)
R² = 0.9859
y = 0.6098x - 0.0278 (□)
R² = 0.9986
y = 0.385x - 0.0345 (△)
R² = 0.9948
(b) Active tillering
y = 0.9277x + 0.431 (○)
R² = 0.9512
y = 0.516x + 0.2157 (□)
R² = 0.9842
y = 0.4294x - 0.1152 (△)
R² = 0.9921
(c) Active tillering
y = 1.0345x + 0.0551 (○)
R² = 0.9661
y = 0.5555x - 0.0047 (□)
R² = 0.9919
y = 0.4101x - 0.0154 (△)
R² = 0.9947
(d) Mid tillering
y = 1.0217x - 1.4813 (○)
R² = 0.9897
y = 0.6032x - 0.9738 (□)
R² = 0.996
y = 0.3805x + 0.3872 (△)
R² = 0.9973
(e) Mid tillering
y = 0.8064x + 3.4089 (○)
R² = 0.9505
y = 0.6017x + 0.2829 (□)
R² = 0.9876
y = 0.3868x - 0.0011 (△)
R² = 0.9849
(f) Mid tillering
y = 0.8992x + 0.2482 (○)
R² = 0.989
y = 0.6042x - 0.4669 (□)
R² = 0.9946
y = 0.3829x + 0.2842 (△)
R² = 0.993
(g) Stem elongation
y = 1.0709x - 14.157 (○)
R² = 0.9597
y = 0.6427x + 0.0505 (□)
R² = 0.9987
y = 0.3582x - 0.3272 (△)
R² = 0.9974
(h) Stem elongation
y = 0.7798x - 0.7437 (○)
R² = 0.982
y = 0.6192x + 0.0536 (□)
R² = 0.996
y = 0.3652x + 0.1376 (△)
R² = 0.9947
(i) Stem elongation
y = 0.8939x - 8.1668 (○)
R² = 0.9806
y = 0.6292x - 0.0769 (□)
R² = 0.9982
y = 0.3617x - 0.0152 (△)
R² = 0.9978
(j) Panicle initiation
y = 0.8919x - 0.4664 (○)
R² = 0.994
y = 0.6631x - 0.9483 (□)
R² = 0.9991
y = 0.3327x + 0.4803 (△)
R² = 0.9974
(k) Panicle initiation
y = 0.5874x + 2.2541 (○)
R² = 0.975
y = 0.6371x - 2.9326 (□)
R² = 0.9959
y = 0.3432x + 1.5852 (△)
R² = 0.9957
(l) Panicle initiation
y = 0.7103x - 0.6007 (○)
R² = 0.9936
y = 0.6482x - 2.1566 (□)
R² = 0.9982
y = 0.3385x + 1.147 (△)
R² = 0.9978
(m) Booting
y = 0.8564x + 0.4104 (○)
R² = 0.9762
y = 0.6117x - 0.1726 (□)
R² = 0.9984
y = 0.3818x - 0.1099 (△)
R² = 0.9985
(n) Booting
y = 0.6417x - 0.8269 (○)
R² = 0.9806
y = 0.6524x + 0.1058 (□)
R² = 0.9981
y = 0.3302x + 0.4523 (△)
R² = 0.9961
(o) Booting
y = 0.7408x - 2.8237 (○)
R² = 0.9954
y = 0.6346x + 0.3133 (□)
R² = 0.9991
y = 0.3517x - 0.129 (△)
R² = 0.9985
(p) Heading
y = 0.6829x - 2.9882 (○)
R² = 0.9865
y = 0.5786x + 2.1011 (□)
R² = 0.9978
y = 0.4022x - 2.0332 (△)
R² = 0.9969
(q) Heading
y = 0.6025x - 3.9376 (○)
R² = 0.9837
y = 0.6085x + 0.0234 (□)
R² = 0.9947
y = 0.3663x - 0.303 (△)
R² = 0.9971
(r) Heading
y = 0.6456x - 4.9045 (○)
R² = 0.992
y = 0.5939x + 1.374 (□)
R² = 0.9978
y = 0.3834x - 1.4652 (△)
R² = 0.9979
260
200
140
80
20
-40
-40 45 130 215 300 385
Nitrogen requirement (NR, kg N ha⁻¹) on LAI/LDM and SDM basis
Nitrogen requirement (NR, kg N ha⁻¹) on PDM basis

Supplementary Fig. 6 The relationships between plant dry matter based nitrogen requirement (NR, kg ha^{-1}) and leaf area index, leaf dry matter and stem dry matter based NR at different growth stages (a, b, c: active tillering; d, e, f: mid tillering; g, h, i: stem elongation; j, k, l: panicle initiation; m, n, o: booting; p, q, r: heading stages) for Japonica, Indica, and Japonica + Indica rice, respectively.
